# Effect of Coal Mining on Health Outcomes Between Male and Female Miners in Southern West Virginia: A Brief Report

**DOI:** 10.7759/cureus.49009

**Published:** 2023-11-18

**Authors:** Frank H Annie, Cassidy Crews, Kerry Drabish, Sangeeta Mandapaka

**Affiliations:** 1 Cardiology, Charleston Area Medical Center (CAMC), Charleston, USA; 2 Research, Charleston Area Medical Center (CAMC), Charleston, USA

**Keywords:** female, male, health outcomes, west virginia, coal miners

## Abstract

Purpose: There is evidence of an association between coal mining and an increased prevalence of respiratory and cardiovascular disease (CVD). Mining is significantly associated with elevated chronic CVD mortality rates. Research is limited and looks at the differences between specific health outcomes between male and female coal miners. The aim of this study was to compare the long-term health outcomes of male and female coal miners in southern West Virginia.

Methods: We used the Charleston Area Medical Center (CAMC) data registry to look at specific health outcomes of coal miners. We queried the data warehouse from September 1, 2016, to January 1, 2023, to identify any coal miners coming to CAMC for any treatment. We identified adult patients aged 18-90 years with at least one visit to a clinic in the CAMC system.

Findings: We identified (n=2,460) cases of coal miners, comprising of 2,280 males and 180 females. Overall, we found higher mortality rates as well as higher rates of ischemic heart disease, heart failure, cancer, and mental health disorders among male coal miners.

Conclusions: Additional research is needed to further examine possible contributing factors that explain the differences in health outcomes between male and female coal miners. Clinicians and policymakers need to address health disparities and occupational hazards that impact the health outcomes of coal miners living in southern West Virginia.

## Introduction

Particulate matter air pollution, like that found in and around coal mines [1], has been shown to increase the risk of morbidity and mortality [2]. There is evidence of an association between coal mining and the increased prevalence of respiratory disease and cardiovascular disease (CVD) [3]. Mining is significantly associated with elevated chronic CVD mortality rates [4]. Coal miners face an increased risk of developing CVD and pulmonary issues because of coal dust and related health care factors [2,5]. Mining is associated with pulmonary diseases, including coal workers’ pneumoconiosis (CWP) and progressive massive fibrosis (PMF), which are found to be more common among underground coal miners [6]. Coal miners are also at risk of developing obstructive and restrictive airway diseases; chronic obstructive pulmonary disease (COPD) is now considered an occupational dust-related disease [7]. In addition, there is an increased risk of cancer mortality, particularly digestive cancers, thyroid cancer, and lung cancer, among miners and those living in the vicinity of mines [8]. Finally, research has shown that the hazards of working environments, including exposure to toxins [9], can be associated with sleep disorders and social issues, both of which impact mental health [10]. Associations have been found between mining and higher rates of depression, anxiety, and substance use disorders [11].

Coal miners in southern West Virginia (WV) face additional barriers, including limited access to primary care and advanced health care in their communities [12]. West Virginia’s rural patterns and economic challenges have led to unequal access to care and “healthcare deserts” [13]. West Virginians experience decreased access to primary care, mental health services, and inpatient facilities. These health disparities contribute to the increased prevalence of cardiovascular disease, heart attacks, cancer, and mental health disorders [14]. WV is ranked highest in the United States in the prevalence of cardiovascular disease; WV is ranked highest in the nation for myocardial infarction and coronary heart disease; and it is ranked fourth in the nation in the prevalence of stroke [14]. WV is ranked sixth in the overall prevalence of cancer, with approximately one in seven West Virginians having been diagnosed with some form of cancer [14]. Finally, WV ranks highest in the nation for adults with depression [14].

In WV, between surface mines and underground mines, 11,333 miners are employed by WV mines [15]. However, studies are limited that examine health outcomes specific to coalminers. In addition, although more men than women work in underground coal mines, men and women are both at increased risk for cardiovascular, kidney, and respiratory disease [16]. Current literature lacks a robust description of the health outcomes specific to each sex, particularly the rates of disease between male and female coal miners. The aim of this study was to compare the long-term health outcomes of male and female coal miners in southern WV. 

## Materials and methods

Study design

This study was a retrospective study with the aim of comparing the long-term health outcomes of male and female coal miners in southern WV.

Inclusion and exclusion criteria

The sample consists of coal mine workers. Inclusion criteria included adult patients aged 18-90 years with at least one visit to a clinic in the CAMC system. Exclusion criteria included those patients outside the specified age range, those not identified as coal miners, and those who had not had at least one visit to a clinic in the CAMC system.

Data collection

We used the CAMC data registry to look at the specific health outcomes of coal miners in southern West Virginia. We queried the data warehouse from September 1, 2016, to January 1, 2023, to identify any coal miners coming to CAMC for any treatment [17]. The data was collected in an MS Excel (Redmond, USA) spreadsheet in preparation for analysis.

Data analysis

Patients were divided into male and female cohorts. These cohorts were analyzed for differences in mortality, ischemic heart disease, heart failure, cancer diagnosis, and mental health disorders. Descriptive statistics were used to measure associations and to create Kaplan-Meier survival curves to assess the endpoints [17]. STATA 11.2 was used to conduct a multinomial logistic regression [17].

## Results

We identified (n=2,460) cases of coal miners that had sought care at CAMC. There were 2,280 males and 180 females. The average age of male coal miners was higher (61.8 ± 16.9 vs. 43.4 ± 17.7, P<0.001) compared to female coal miners [17] (Table 1).

**Table 1:** Demographics

N=2460	Cohort	Percentage	Age
Male	n=2280	92.7%	61.8±16.9
Female	n=180	7.3%	43.4±17.7

We found that male coal miners had a higher mortality rate (8.3% vs. 5.5%, P=0.19) compared to females; a log rank test was 88.6% vs. 92.7% (P=0.03) at three years [17]. Ischemic heart disease was higher in males (40.3% vs. 16.6%, P<0.001) compared to females; a log rank test was 46.9% vs. 78.2% (P<0.001) at three years [17]. Heart failure was also higher in the male coal miners (17.9% vs. 5.5%, P<0.001), with a log rank of 75.8% vs. 93.4%) P<0.001) [17]. Cancer rates were also higher in the male group, 40.3% vs. 33.3% (P=0.06), with a log rank of 44.2% vs. 51.3% (P=0.01) [17]. Mental health disorders were lower in female coal miners (34.2% vs. 55.5%, P<0.001) compared to male coal miners; a log rank test was 53.6% vs. 29.6%, P<0.001) [17] (Table 2).

**Table 2:** Comparison of Outcomes

Outcome	Mortality	Ischemic Heart Disease	Heart Failure	Cancer Rates	Mental Health Disorders
Male	8.3%	40.3%	17.9%	40.3%	34.2%
Female	5.5%	16.6%	5.5%	33.3%	55.5%
P value	0.19	<0.001	<0.001	0.06	<0.001
Log Rank Test	Mortality	Ischemic Heart Disease	Heart Failure	Cancer Rates	Mental Health Disorders
Male	88.6%	46.9%	75.8%	44.2%	53.6%
Female	92.7%	78.2%	93.4%	51.3%	29.6%
P value	0.03	<0.001	<0.001	0.01	<0.001

## Discussion

The results of our study demonstrate higher mortality rates as well as higher rates of ischemic heart disease, heart failure, cancer, and mental health disorders among male coal miners. The findings of this study align with current research on the health status of coal miners and individuals living in Appalachian regions. For example, Hendryx and associates not only found poorer health outcomes in mining areas, but they also discovered that as coal production increased, rates of disease increased [17]. Zullig and Hendryx found that residents of coal-mining counties reported fewer days of good physical and mental health [1]. Landon and associates found that even after adjusting for confounding variables, coal dust exposure was associated with an increased risk of ischemic heart disease mortality [2]. In addition, after adjusting for covariates, Esch and Hendryx found that mortality rates were higher in mountain top mining communities [4]. A study by Fernández-Navarro and associates found that men and women living near underground mining facilities demonstrated increased mortality rates related to bladder, colorectal, and lung cancers [8].

Limitations of our study include a limited sample of female coal miners. However, our sample is representative of the overall lower number of female coal miners in WV. Our results demonstrate lower rates of mortality as well as lower rates of ischemic heart disease, heart failure, cancer, and mental health disorders among female coal miners; however, a larger sample may yield different results. The age differences between male and female coal miners may account for some differences in rates of disease. In addition, we did not account for confounding variables such as tobacco use, alcohol use, family health histories, or diet; certainly, these factors could influence health outcomes.

Additional research is needed to determine whether the differences in outcomes stem from a difference in the utilization of healthcare services. Subsequent studies may examine the health outcomes of male and female coal miners in relation to their proximity to health care facilities as well as the frequency of health-related visits. Shifting focus to implementing changes that will reduce health disparities experienced by those in southern West Virginia is a necessary first step in promoting optimum health outcomes for coal miners and their communities. In addition, maintaining health clinics at mining sites to provide acute and preventative care and health education could go a long way toward coal miners’ health promotion [16].

## Conclusions

Coal miners in southern West Virginia experience health disparities and are at increased risk of ischemic heart disease, heart failure, cancer, and mental health disorders. We sought to explore the differences between health outcomes among male and female coal miners. Although our results show that male coal miners experience an increased prevalence of these health outcomes, additional research is needed to confirm our findings and explore additional factors impacting the health of coal miners in southern West Virginia. This study is a necessary first step in attempting to reduce health disparities and improve the health of coal miners and their communities.
